# Correction: Impact of PARP inhibitor maintenance therapy in newly diagnosed advanced epithelial ovarian cancer: A meta-analysis

**DOI:** 10.1371/journal.pone.0317158

**Published:** 2025-01-03

**Authors:** Banghyun Lee, Suk-Joon Chang, Byung Su Kwon, Joo-Hyuk Son, Myong Cheol Lim, Yun Hwan Kim, Shin-Wha Lee, Chel Hun Choi, Kyung Jin Eoh, Jung-Yun Lee, Dong Hoon Suh, Yong Beom Kim

The Funding statement for this paper is incorrect. The correct Funding statement is as follows: This work was supported by the Research Fund of the National Cancer Center, Korea, Republic of (NCC-2112570-3).
