# Supplementary material for: Impact of PARP inhibitor maintenance therapy in newly diagnosed advanced epithelial ovarian cancer: A meta-analysis
Source: PLoS One. 2023 Nov 17;18(11):e0294647. doi: 10.1371/journal.pone.0294647 (PMC10655973; doi:10.1371/journal.pone.0294647)

S1 Fig. Assessments of the risk of bias in the included studies.

(A) Two-year PFS

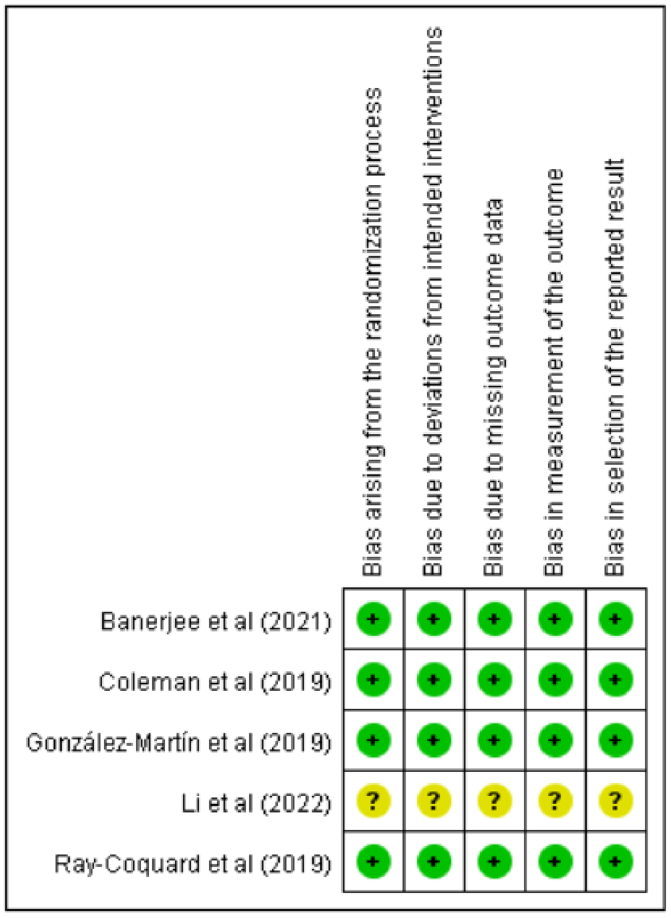

(B) Five-year OS

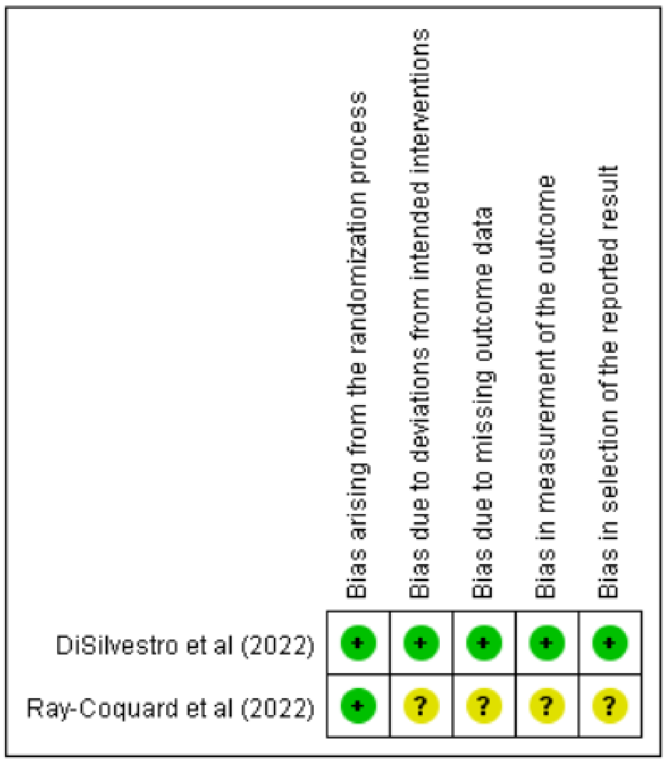

Low risk
Unclear risk

(C) Adverse events ≥ grade 3

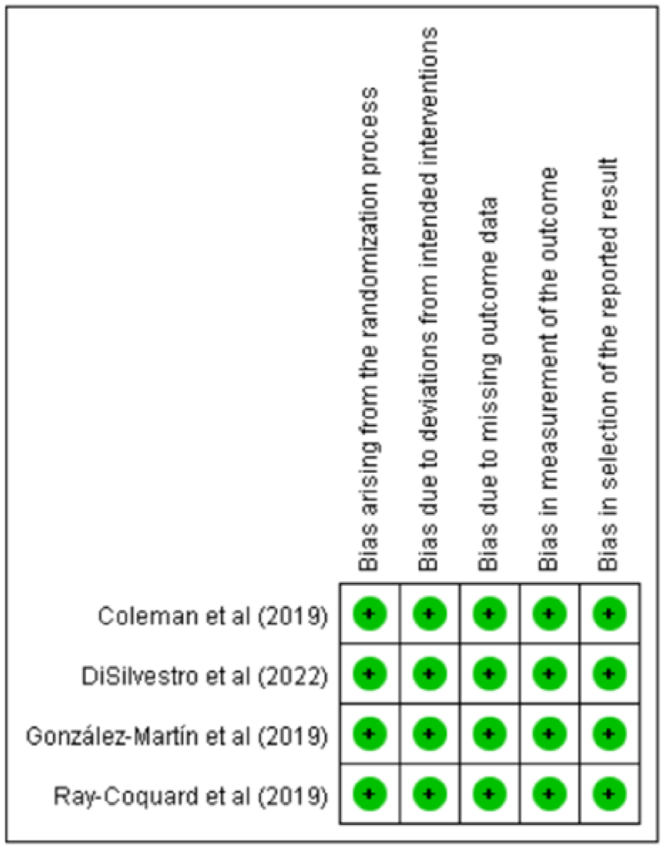

Supplement: S1 Fig — (A) two-year PFS, (B) five-year OS, and (C) Adverse events ≥ grade 3. (PDF) [file pone.0294647.s002.pdf]
