# Supplementary material for: Impact of PARP inhibitor maintenance therapy in newly diagnosed advanced epithelial ovarian cancer: A meta-analysis
Source: PLoS One. 2023 Nov 17;18(11):e0294647. doi: 10.1371/journal.pone.0294647 (PMC10655973; doi:10.1371/journal.pone.0294647)
Supplement: S1 Table — (A) Pubmed, (B) Cochrane Library, (C) Embase, and (D) KoreaMed. (PDF) [file pone.0294647.s003.pdf]

Supplementary Table S1. Search strategy.

Pubmed

Search number	Query	Results
#1	ovary [TW] OR ovari* [TW] OR fallopian tube* [TW] OR Fallopian tubal [TW] OR salpinx [TW] OR peritoneum [TW] OR peritoneal [TW] OR adnexa*[TW] OR ovary [MH] OR fallopian tubes [MH] OR peritoneum[MH]	524,751
#2	cancer* [TW] OR carcinoma* [TW] OR neoplasm* [TW] OR adenocarcinoma*[TW] OR malignan*[TW] OR Neoplasms[MH] OR Carcinoma[MH] OR Adenocarcinoma[MH]	4,582,643
#3	Ovarian neoplasms [MH] OR Carcinoma, Ovarian Epithelial [MH] OR fallopian tube neoplasms [MH] OR peritoneal neoplasms[MH]	109,115
#4	(#1 AND #2) OR #3	216,376
#5	"Poly ADP-ribose Polymerase Inhibitors" [MH] OR Poly ADP-Ribose Polymerase Inhibitor* [TW] OR "Poly ADP-Ribose Polymerase" [TW] OR Poly ADP-ribosylation Inhibitor* OR poly adenosine diphosphate [ADP]-ribose polymerase inhibitor* OR PARP Inhibitor* OR PARP [TW] OR PARPi* OR olaparib [TW] OR rucaparib [TW] OR veliparib [TW] OR niraparib [TW] OR talazoparib [TW] OR iniparib [TW] OR pamiparib [TW] OR lynparza [TW] OR rubraca [TW] OR zejula [TW] OR talzenna [TW]	30,961
#6	Surviv*[TW] OR survival [MH] OR Progression-Free Survival [MH] OR "Progression-Free Survival" [TW] OR PFS [TIAB] OR Disease-Free Survival [MH] OR "Disease-Free Survival" [TW] OR DFS [TIAB] OR event-free survival [TW] OR overall survival* [TW] OR OS [TIAB] OR Mortality [MH] OR Mortalit*[TW] OR death [MH] OR death* [TW] OR prognos*[TW]	3,833,563
#7	#4 AND #5 AND #6	1,531
#8	#7 NOT (review[PT] OR review Literature as topic[MH])	1,089
#9	#8 NOT (animals[MH] NOT (humans[MH] AND animals[MH]))	1,072

Cochrane Library

Search number	Query	Results

#1	((ovary OR ovari* OR fallopian tube* OR Fallopian tubal OR salpinx OR peritoneum OR peritoneal OR adnexa*):ti,ab,kw OR ([mh ovary] OR [mh peritoneum] OR [mh "Fallopian tubes"])) NEAR/3 ((cancer* OR carcinoma* OR neoplasm* OR adenocarcinoma* OR malignan*):ti,ab,kw OR ([mh Neoplasms] OR [mh Carcinoma] OR [mh Adenocarcinoma]))	8,858
#2	[mh "ovarian neoplasms"] OR [mh "Carcinoma, Ovarian epithelial"] OR [mh "Fallopian Tube Neoplasms"] OR [mh "Peritoneal Neoplasms"]	2,435
#3	#1 OR #2	8,897
#4	[mh "Poly(ADP-ribose) Polymerase Inhibitors"] OR (Poly ADP-Ribose Polymerase Inhibitor* OR "poly ADP ribose polymerase" OR poly adenosine diphosphate-ribose polymerase inhibitor* OR PARP Inhibitor* OR PARP OR PARPi* OR olaparib OR rucaparib OR veliparib OR niraparib OR talazoparib OR iniparib OR pamiparib OR lynparza OR rubraca OR zejula OR talzenna):ti,ab,kw	1,624
#5	[mh "survival"] OR [mh "Progression-Free Survival"] OR [mh "Disease-Free Survival"] OR [mh Mortality] OR [mh death] OR (Surviv* OR "Progression-Free Survival" OR PFS OR "Disease-Free Survival" OR DFS OR "event-free survival" OR overall survival* OR OS OR Mortalit* OR death* OR prognos*):ti,ab,kw	268,581
#6	#3 AND #4 AND #5	520
#7	#6 NOT ((review):pt OR [mh "Review Literature as Topic"])	520
#8	#7 NOT ([mh "animals"] NOT ([mh "humans"] AND [mh "animals"]))	520

Embase

Search number	Query	Results
#1	((ovar* OR 'fallopian tub*' OR salpinx OR peritoneum OR peritoneal OR adnexa*) NEAR/3 (cancer* OR carcinoma* OR neoplasm* OR adenocarcinoma* OR malignan*)):ti,ab,kw	146,555
#2	'ovary cancer'/exp OR 'ovary carcinoma'/exp OR 'ovary adenocarcinoma'/exp	139,168
#3	#1 OR #2	187,705

#4	'survival'/exp OR 'progression free survival'/exp OR 'disease free survival'/exp OR 'mortality'/exp OR 'death'/exp OR surviv*:ti,ab,kw OR 'progression free survival':ti,ab,kw OR pfs:ti,ab,kw OR 'disease free survival':ti,ab,kw OR dfs:ti,ab,kw OR 'event free survival':ti,ab,kw OR 'overall survival':ti,ab,kw OR os:ti,ab,kw OR mortalit*:ti,ab,kw OR death*:ti,ab,kw OR prognos*:ti,ab,kw	5,231,625
#5	'nicotinamide adenine dinucleotide adenosine diphosphate ribosyltransferase inhibitor'/exp OR 'poly adp-ribose polymerase inhibitor*':ti,ab,kw OR 'poly adenosine diphosphate-ribose polymerase inhibitor*':ti,ab,kw OR 'parp inhibit*':ti,ab,kw OR parpi*:ti,ab,kw OR olaparib:ti,ab,kw OR rucaparib:ti,ab,kw OR veliparib:ti,ab,kw OR niraparib:ti,ab,kw OR talazoparib:ti,ab,kw OR iniparib:ti,ab,kw OR pamiparib:ti,ab,kw OR lynparza:ti,ab,kw OR rubraca:ti,ab,kw OR zejula:ti,ab,kw OR talzenna:ti,ab,kw	22,478
#6	#3 AND #4 AND #5	3,692
#7	#6 NOT (review:it OR 'literature'/exp)	2,715
#8	#7 NOT ('animal'/exp NOT ('human'/exp AND 'animal'/exp))	2,642

KoreaMed

Search number	Query	Results
#1	((ovarian[ALL] OR Ovary[ALL] OR fallopian[ALL] OR Peritoneal[ALL] OR Peritoneum[ALL] OR salpinx[ALL] OR Adnexal[ALL] OR ("ovarian neoplasms"[MH]) OR ("carcinoma, ovarian epithelial"[MH]) OR ("fallopian tube neoplasms"[MH]) OR ("peritoneal neoplasms"[MH])))	7,067
#2	Poly[ALL] OR PARP[ALL] OR olaparib[ALL] OR rucaparib[ALL] OR veliparib[ALL] OR niraparib[ALL] OR talazoparib[ALL] OR iniparib[ALL] OR pamiparib[ALL] OR lynparza[ALL] OR rubraca[ALL] OR zejula[ALL] OR talzenna[ALL]	752

#3	(("ovarian"[ALL]) OR ("Ovary"[ALL]) OR ("fallopian"[ALL]) OR ("Peritoneal"[ALL]) OR ("Peritoneum"[ALL]) OR ("salpinx"[ALL]) OR ("adnexal"[ALL]) OR ("ovarian neoplasms"[MH]) OR ("carcinoma, ovarian epithelial"[MH]) OR ("fallopian tube neoplasms"[MH]) OR ("peritoneal neoplasms"[MH])) AND (Poly[ALL] OR PARP[ALL] OR olaparib[ALL] OR rucaparib[ALL] OR veliparib[ALL] OR niraparib[ALL] OR talazoparib[ALL] OR iniparib[ALL] OR pamiparib[ALL] OR lynparza[ALL] OR rubraca[ALL] OR zejula[ALL] OR talzenna[ALL])		46
