# Supplementary material for: Impact of PARP inhibitor maintenance therapy in newly diagnosed advanced epithelial ovarian cancer: A meta-analysis
Source: PLoS One. 2023 Nov 17;18(11):e0294647. doi: 10.1371/journal.pone.0294647 (PMC10655973; doi:10.1371/journal.pone.0294647)
Supplement: S2 Table — CI: confidence interval; HR: hazard ratio; HRD: homologous-recombination deficiency; HRP: homologous-recombination proficiency; OR: odds ratio; OS: overall survival; PARPi: poly (adenosine diphosphate [ADP]–ribose) polymerase inhibitors; PFS: progression-free survival; RCT: randomized controlled studies. a. High heterogeneity unexplained, b. Wide confidence interval that crosses 1 and is out of the area between 0.75 and 1.25. (PDF) [file pone.0294647.s004.pdf]

S2 Table. GRADE evidence profiles.

Quality assessment							№ of patients		Effect		Quality
**№ of studies**	**Study design**	**Risk of bias**	**Inconsistency**	**Indirectness**	**Imprecision**	**Publication Bias**	**PARPi**	**No PARPi**	**Relative (95% CI)**	**Absolute (95% CI)**	
**2-year PFS (all population)**											
5	randomised trials	not serious	serious[a]	not serious	not serious	none	1921 participants	1150 participants	**HR 0.53** (0.41 to 0.68)	**225 fewer per 1,000** (from 316 fewer to 133 fewer)	⊕⊕⊕◯ Moderate
**2-year PFS (Patients with BRCAm)**											
4	randomised trials	not serious	not serious	not serious	not serious	none	677 participants	372 participants	**HR 0.35** (0.29 to 0.42) [2-year PFS (BRCA mutation)]	**339 fewer per 1,000** (from 384 fewer to 291 fewer)	⊕⊕⊕⊕ High
**2-year PFS (Patients with BRCA wild type)**											
2	randomised trials	not serious	not serious	not serious	not serious	none	625 participants	443 participants	**HR 0.75** (0.65 to 0.88) [2-year PFS (BRCA wild type)]	**105 fewer per 1,000** (from 157 fewer to 46 fewer)	⊕⊕⊕⊕ High
**2-year PFS (Patients with HRD)**											
3	randomised trials	not serious	not serious	not serious	not serious	none	716 participants	465 participants	**HR 0.44** (0.32 to 0.60) [2-year PFS (HRD)]	**277 fewer per 1,000** (from 360 fewer to 181 fewer)	⊕⊕⊕⊕ High
**2-year PFS (Patients with HRD without BRCAm)**											
3	randomised trials	not serious	not serious	not serious	not serious	none			**HR 0.58** (0.38 to 0.89) [2-year PFS (HRD without BRCA mutation)]	**-- per 1,000** (from -- to --)	⊕⊕⊕⊕ High

Quality assessment							№ of patients		Effect		Quality
**№ of studies**	**Study design**	**Risk of bias**	**Inconsistency**	**Indirectness**	**Imprecision**	**Publication Bias**	**PARPi**	**No PARPi**	**Relative (95% CI)**	**Absolute (95% CI)**	
**2-year PFS (Patients with HRP)**											
3	randomised trials	not serious	not serious	not serious	serious[b]	none	486 participants	289 participants	**HR 0.83** (0.66 to 1.03) [2-year PFS (HRP)]	**67 fewer per 1,000** (from 151 fewer to 10 more)	⊕⊕⊕◯ Moderate
**5-year OS (all population)**											
2	randomised trials	not serious	serious[a]	not serious	serious[b]	none	797 participants	400 participants	**HR 0.73** (0.44 to 1.20)	**107 fewer per 1,000** (from 251 fewer to 66 more)	⊕⊕◯◯ Low
**5-year OS (Patients with BRCAm)**											
2	randomised trials	not serious	not serious	not serious	not serious	none	417 participants	211 participants	**HR 0.57** (0.44 to 0.74) [5-year OS (BRCA mutation)]	**170 fewer per 1,000** (from 231 fewer to 97 fewer)	⊕⊕⊕⊕ High
**Adverse event (Grade≥3)**											
4	randomised trials	not serious	serious[a]	not serious	not serious	none	1079/1656 (65.2%)	493/1012 (48.7%)	**OR 2.94** (1.13 to 7.63)	**249 more per 1,000** (from 31 more to 392 more)	⊕⊕⊕◯ Moderate

CI: confidence interval; HR: hazard ratio; HRD: homologous-recombination deficiency; HRP: homologous-recombination proficiency; OR: odds ratio; OS: overall survival; PARPi: poly (adenosine diphosphate [ADP]–ribose) polymerase inhibitors; PFS: progression-free survival; RCT: randomized controlled studies.

a. High heterogeneity unexplained, b. Wide confidence interval that crosses 1 and is out of area between 0.75 and 1.25.
